# Editorial: Genomics assisted improvement of crop plants for adaptation to marginal environments

**DOI:** 10.3389/fgene.2024.1461709

**Published:** 2024-07-22

**Authors:** Prashant Vikram, Sajid Shokat, Amita Mohan, Deepmala Sehgal, Mayank Kashyap

**Affiliations:** ^1^ Director Research, Faculty of Agricultural Science, Shree Guru Gobind Singh Tricentenary University, Gurugram, Haryana, India; ^2^ Plant Breeding and Genetics Laboratory, International Atomic Energy Agency Laboratories, Seibersdorf, Austria; ^3^ Nuclear Institute for Agriculture and Biology, Faisalabad, Pakistan; ^4^ Department of Biology, University of Pennsylvania, Philadelphia, PA, United States; ^5^ Syngenta, Bracknell, United Kingdom; ^6^ Agriculture Victoria, Melbourne, VIC, Australia

**Keywords:** genomics assisted breeding (GAB), heat tolerance, drought tolerance, disease resistance, grain quality

Meeting the food security demands of 9 billion people by 2050 is a monumental task that necessitates the development of future crops facing the challenges of climate change on various fronts. Nearly three billion people reside in regions with moderate to acute water shortages, poor quality water, inferior soil health (salt stress), and low-input rain-fed farming systems (FAO 2020). Thus, improving crop resilience and adaptation to marginal areas will be crucial in reaching sustainable agricultural goals. Geneticists and plant breeders have benefited immensely from recent advancements in genomic tools, laying the foundation for modern plant breeding solutions. Research centres and agricultural programs have launched massive efforts to develop high-yielding climate-resilient crops. This research topic aimed to enhance cooperation amongst groups working on improving the genetics of the crops for marginal lands and to share state-of-the-art findings with the scientific community, thereby promoting creative, sustainable food production with minimal impact on the soil.

The initial studies emphasized wheat’s resistance to abiotic stress; Taria et al. used a multivariate analysis approach using RIL population to identify QTLs associated with SPAD value, leaf senescence rate (LSR), and stem reserve mobilization efficiency (SRE). Similarly, Manjunath et al. improved our knowledge of genetic responses to drought by identifying critical yield-related hotspots on chromosomes 6B and 5B in wheat under moisture stress. With temperature rising across different regions, it becomes imperative to decipher its impact and mitigating strategies. In their evaluation of the most recent omics techniques, Ijaz et al. highlighted possible avenues for improving crop tolerance to high temperatures while shedding light on cotton genetic and molecular mechanisms underlying heat stress. These reports indicate that applying high-density genomics accompanied by under-utilized germplasm can be the way forward to improving the crops for marginal environments.

Another interesting study by Laribi et al. with a set of Tunisian durum wheat landrace identified genomic regions associated with the tan spot disease (*Pyrenophora tritici-repentis*). The authors conducted genome-wide association mapping and identified regions explaining 8.1%–20.2% phenotypic variation along with eight resistant donor accessions carrying positive alleles for further application in genomics-assisted breeding. Study presents the successful deployment of high-density genomic applications for under-utilized valuable germplasms. The authors also utilized comparative genomics and identified conserved domains in genes associated with disease resistances. In *Brassica oleracea*, Sadaqat et al. using the genome-wide identification and expression profiling of two-component system (TCS) genes and found 80 BoTCS genes. The ability of these genes to change their expression successfully under shade stress suggest that they are crucial for biological processes. The initial description of the CC-NBS-LRR (CNL) gene family in purple (*Passiflora edulis* Sims.) and yellow (*Passiflora edulis f. flavicarpa*) passion fruits was published in 2024 by Zia et al. Similarly, by a pangenome analysis in eight Pyrus genomes, Yang et al. provided the first report of the transport inhibitor Response1/Auxin signalling F-box (TIR1/AFB). The comparative genomics investigation of these family’s genes further revealed their involvement in fruit hardening and drought stress mitigation. It was discovered that these genes are dispersed across 17 chromosomes. Further, reports related to the biotic stress resistance in this issue emphasize the importance of conserved domains and motifs of the disease resistance genes (i.e., R genes). Studies also indicate that the application of comparative genomics in biotic stress resistance is needed at a large scale. Identification, characterization, and deployment of R genes through genetic engineering tools, including gene editing, can bring a paradigm shift in biotic stress mitigation. Change in disease patterns given climate change in the marginal areas presents a major challenge that can be tackled efficiently using a multitude of approaches, including comparative genomics.

Other than genetic background, environmental conditions in marginal areas affect the grain quality. Several studies have been conducted to determine the possibility of genomic selection in grain quality parameters for use in breeding programs. For example, Ndlovu et al., recently conducted a linkage mapping investigation in maize (*Zea mays* L.) for grain quality traits. It is noteworthy that certain traits cannot be handled effectively using conventional approaches. Thus, genomic selection should be pursued to handle the breeder’s bottleneck traits, i.e., for which breeders cannot perform selection conventionally. The efficiency of genomic selection can be improved, focusing on accumulating major effect positive alleles in the training/test population.

In summary, the success of genomics-assisted crop improvement for marginal areas largely depends on the approach, tool, type of germplasm used, and scale of efforts. Based on the success achieved so far, the approaches and tools can be decided. Germplasm type should focus on the accumulation of positive alleles as well as the broadening of genetic bases, and finally, the scale of effort must be designed in a way that does not lose the positive alleles of germplasm used. Therefore, a comprehensive strategy is required, blending various methods and techniques for achieving genetic gains in crop varieties to adapt to marginal environments.

